# Elevated triglyceride-glucose-body mass index associated with lower probability of future regression to normoglycemia in Chinese adults with prediabetes: a 5-year cohort study

**DOI:** 10.3389/fendo.2024.1278239

**Published:** 2024-02-13

**Authors:** Yang Shao, Haofei Hu, Changchun Cao, Yong Han, Cen Wu

**Affiliations:** ^1^ Department of Laboratory Medicine, Shengjing Hospital of China Medical University, Shenyang, Liaoning, China; ^2^ Liaoning Clinical Research Center for Laboratory Medicine, Shenyang, Liaoning, China; ^3^ Department of Nephrology, Shenzhen Second People’s Hospital, Shenzhen, Guangdong, China; ^4^ Department of Rehabilitation, Shenzhen Dapeng New District Nan’ao People’s Hospital, Shenzhen, Guangdong, China; ^5^ Department of Emergency, Shenzhen Second People’s Hospital, Shenzhen, Guangdong, China; ^6^ Department of Pulmonary and Critical Care Medicine, Shengjing Hospital of China Medical University, Shenyang, Liaoning, China

**Keywords:** prediabetes, conversion to normoglycemia, TyG-BMI, nonlinear, competitive risk model

## Abstract

**Objective:**

Despite the clear association of TyG-BMI with prediabetes and the progression of diabetes, no study to date has examined the relationship between TyG-BMI and the reversal of prediabetes to normoglycemia.

**Methods:**

25,279 participants with prediabetes who had physical examinations between 2010 and 2016 were enrolled in this retrospective cohort study. The relationship between baseline TyG-BMI and regression to normoglycemia from prediabetes was examined using the Cox proportional hazards regression model in this study. Additionally, the nonlinear association between TyG-BMI and the likelihood of regression to normoglycemia was investigated using the Cox proportional hazards regression with cubic spline function. Competing risk multivariate Cox regression analysis was conducted, with progression to diabetes as a competing risk for prediabetes reversal to normoglycemia. Furthermore, subgroup analyses and a series of sensitivity analyses were performed.

**Results:**

After adjusting for covariates, the results showed that TyG-BMI was negatively associated with the probability of returning to normoglycemia (per 10 units, HR=0.970, 95% CI: 0.965, 0.976). They were also nonlinearly related, with an inflection point for TyG-BMI of 196.46. The effect size (HR) for TyG-BMI to the right of the inflection point (TyG-BMI ≥ 196.46) and the probability of return of normoglycemia was 0.962 (95% CI: 0.954, 0.970, per 10 units). In addition, the competing risks model found a negative correlation between TyG-BMI and return to normoglycemia (SHR=0.97, 95% CI: 0.96-0.98). Sensitivity analyses demonstrated the robustness of our results.

**Conclusion:**

This study demonstrated a negative and nonlinear relationship between TyG-BMI and return to normoglycemia in Chinese adults with prediabetes. Through active intervention, the combined reduction of BMI and TG levels to bring TyG-BMI down to 196.46 could significantly increase the probability of returning to normoglycemia.

## Introduction

Prediabetes (Pre-DM) is a condition characterized by elevated blood glucose levels that do not meet the diagnostic criteria for diabetes (DM) (1). It includes two subcategories known as impaired glucose tolerance (IGT) and impaired fasting glucose (IFG), as defined by the American Diabetes Association (ADA) (2). The global prevalence of Pre-DM has been rapidly increasing, with an estimated 536.6 million adults diagnosed in 2021, according to the International Diabetes Federation (IDF). Furthermore, the IDF projects that by 2045, the number of adults with Pre-DM will rise to 783.2 million, comprising approximately 12.2% of the adult population (3). Individuals diagnosed with Pre-DM face an elevated risk of developing DM, as well as an increased risk of cardiovascular disease and microvascular complications. It is estimated that each year, approximately 5-10% of people with Pre-DM progress to DM, and eventually, up to 70% may develop DM (4, 5). However, it is important to note that not all individuals with pre-DM progress to diabetes. Some individuals with Pre-DM may remain in the prediabetic stage without developing DM, and a significant proportion, ranging from 20% to 50%, may even experience a regression to normoglycemia (6–8). Previous studies have provided compelling evidence that even a temporary return to normoglycemia is associated with a substantial reduction in the risk of developing DM and its associated complications among individuals with Pre-DM (9–12). Therefore, achieving a return from Pre-DM to normoglycemia holds significant clinical significance. The primary goal of Pre-DM screening and treatment should be to restore blood glucose levels to within the normal range.

Therefore, it is critical to investigate the prevalence of return to normoglycemia in patients with Pre-DM and to identify the associated contributing factors. This can greatly influence public health initiatives, enabling them to develop effective strategies to prevent the progression of Pre-DM to DM and to improve the long-term health status of at-risk populations (10, 11). Unfortunately, much of the attention on the clinical side appears to be focused on disease progression, with a limited number of studies exploring rates of return to normoglycemia and associated contributing factors in patients with Pre-DM. Preliminary evidence from previous epidemiological studies indicates that several factors may be associated with regression to normoglycemia in individuals with Pre-DM. These factors include age, baseline fasting glucose levels, insulin secretion, body mass index (BMI), beta-cell function, and fasting triglyceride (TG) levels (13–15). Recently, there has been a surge of interest in a marker called triglyceride glucose-body mass index (TyG-BMI). According to Er et al. TyG-BMI has been described as superior to other alternative markers of insulin resistance(IR) in identifying IR (16). In addition, several observational research studies have found good predictive performance in the assessment of hypertension, nonalcoholic fatty liver disease (NAFLD), metabolic syndrome (MS), and hyperuricemia (17–19). Multiple studies have demonstrated a notable positive association between TyG-BMI and both Pre-DM and DM, indicating its potential as a reliable predictor (20–23). However, there is a lack of research examining the relationship between TyG-BMI and the regression to normoglycemia in individuals with Pre-DM. Therefore, the objective of this study was to investigate the relationship between TyG-BMI and the probability of reversal to normoglycemia in the Chinese prediabetic population and to provide new ideas to facilitate the achievement of normoglycemia and the prevention of DM in individuals diagnosed with Pre-DM.

## Methods

### Study design and data source

This *post-hoc* analysis was derived from a cohort study conducted by the esteemed Rich Healthcare Group in China (24). The raw data utilized for the present analysis was graciously provided by Chen et al. (24) and are readily accessible on DATADRYAD (www.datadryad.org). For more detailed information, readers can refer to the published article titled “Association of body mass index and age with the onset of diabetes in Chinese adults: a population-based cohort study” (https://doi.org/10.5061/dryad.ft8750v) (24). This article is published under the Creative Commons Attribution Noncommercial (CC BY-NC 4.0) license, permitting the sharing, republishing, modification, and creation of derivative works of the original material for noncommercial purposes (24).

### Study population

The initial researchers obtained information from a computerized database established by the China Rich Healthcare Group (24). This comprehensive database encompassed all medical records of participants who underwent health screenings from 2010 to 2016, spanning 32 regions and 11 cities across China. The original study provided data for participants with a minimum follow-up of 2 years and a maximum follow-up of 5 years. The Rich Healthcare Group review board gave preliminary approval for the original study and conducted a retrospective search of the information. Due to the study’s retrospective nature, the institutional ethics committee waived the requirement for informed consent and approval (24). As with other studies utilizing this database, this study did not require a new application as the data remains anonymous, and ethical approval for the research was already granted in the original study (21, 24–26).

**Figure 1:**
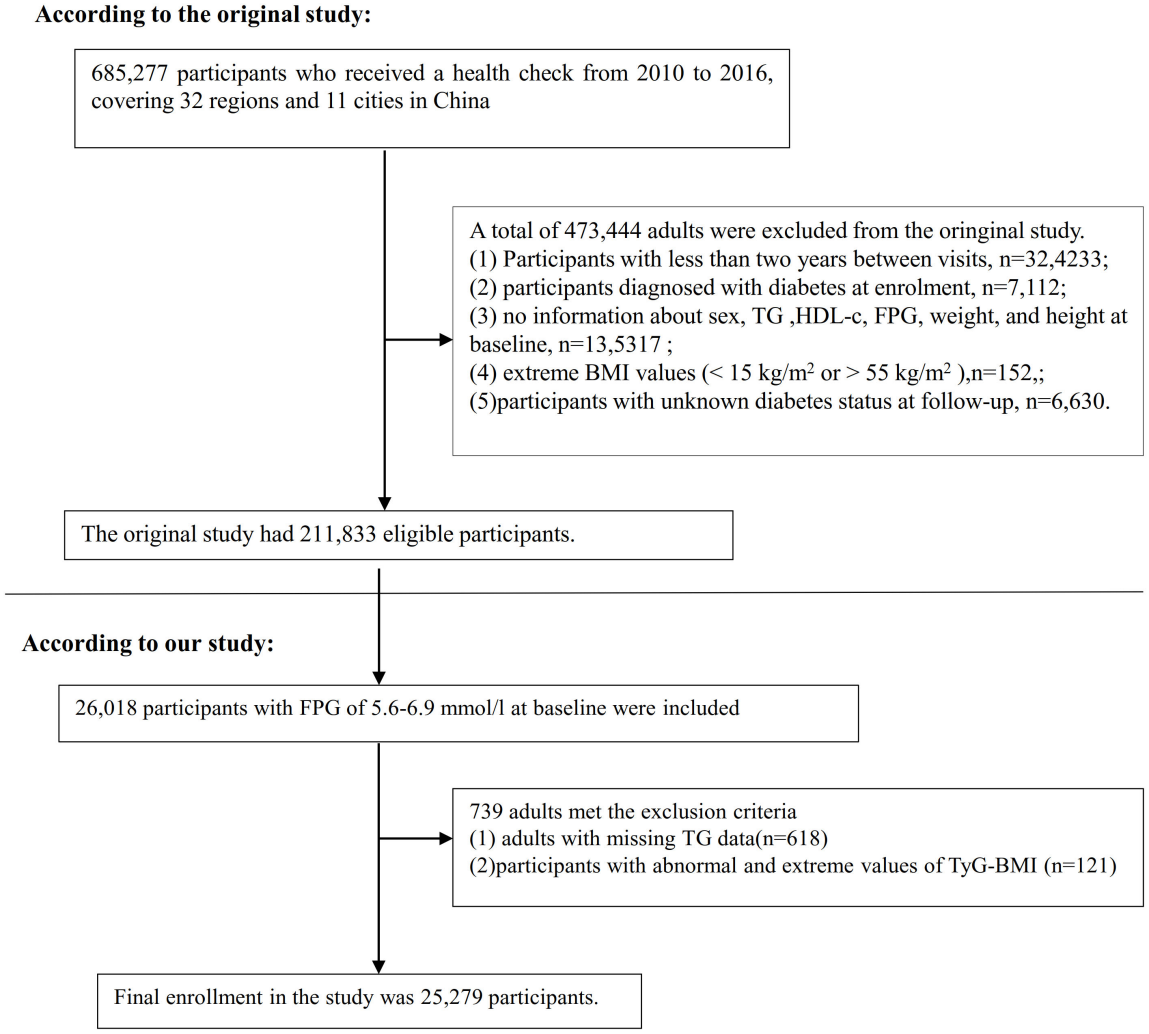
Flowchart illustrating the composition of study participants.

The initial study included a cohort of 685,277 individuals aged 20 years and older who had undergone at least two health screenings. Out of these, 473,444 participants were excluded from the analysis as they did not meet the original study’s exclusion criteria. The exclusion criteria were as follows: (i) individuals with a time interval less than two years between medical examinations; (ii) individuals lacking data on fasting plasma glucose (FPG), body weight, sex, height values at the beginning of the study; (iii) individuals with exceptionally high or low BMI(15 kg/m^2^ or > 55 kg/m^2^); (iv) individuals diagnosed with diabetes at the time of enrollment; (v) individuals with an unknown diabetes status during the follow-up period. In the initial study, the analysis was conducted on a total of 211,833 participants (24). In the present study, 26,018 individuals with prediabetes were incorporated, who exhibited a baseline FPG level ranging from 5.6 to 6.9 mmol/L, in accordance with the diagnostic criteria outlined by the ADA in 2021 (2). Subsequently, 618 participants were excluded from the analysis due to the absence of TG data. Additionally, 317 participants were excluded as they presented with an abnormal or extreme TyG-BMI, defined as values greater or less than three standard deviations from the mean. A total of 25,279 participants were enrolled in the present study. **Figure 1** showed how participants were selected.

### Variables

#### Triglyceride glucose-body mass index calculation

The exact process for defining TyG-BMI in the study was as follows: TyG-BMI was calculated using the formula TyG-BMI = BMI × TyG index, where TyG index = ln [FPG (mg/dL) × TG (mg/dL)/2], and BMI = weight/height^2^ ((kg/m^2^) (16).

#### Reversion to normoglycemia from prediabetes

Regression to normoglycemia in patients with Pre-DM was defined as having an FPG <5.6 mmol/L at the follow-up assessment and the absence of self-reported DM events (2).

### Covariates

The covariates utilized in our study were chosen in accordance with the original study, as well as previous research endeavors pertaining to Pre-DM or DM, and our clinical proficiency. The covariates encompassed the subsequent variables: (i) Continuous variables: weight, diastolic blood pressure (DBP), alanine aminotransferase (ALT), age, low-density lipoprotein cholesterol (LDL-c), serum creatinine (Scr), high-density lipoprotein cholesterol (HDL-c), systolic blood pressure (SBP), aspartate aminotransferase (AST), total cholesterol (TC), blood urea nitrogen (BUN), and height. (ii) Categorical variables include family history of diabetes, smoking status, sex, and drinking status.

### Data collection

A standardized questionnaire was used by trained healthcare professionals to collect baseline data on the subjects, which included their height, sex, weight, family history, age, smoking and drinking status, and other biochemical indicators assessed through physical examination. A typical mercury sphygmomanometer was used to measure blood pressure. After at least 10 hours of fasting, samples were taken for biochemical indices examination, which were then evaluated by a Beckman 5800 biochemical analyzer (24).

### Treatment of missing data

In the second analysis, there were 2439 (9.65%), 7 (0.03%), 211 (0.84%), 7 (0.03%), 9,291 (36.75%), 1,119 (4.43%), 1,4118 (55.85%), 16,727 (66.17%), 9,921 (39.25%), and 16,727 (66.17%) participants with missing data for BUN, SBP, ALT, DBP, LDL-c, Scr, AST, drinking status, HDL-c, and smoking status. Multiple interpolation was utilized in this study for missing data to reduce the variation caused by missing variables (27). The TC, drinking status, gender, HDL-c, DBP, age, BUN, Scr, SBP, ALT, family history of diabetes, LDL-c, AST, and smoking status were all included in the estimation model (linear regression with ten iterations). The missing at random (MAR) premise was applied to the missing data analysis process (27, 28).

### Statistical analyses

Statistical analyses were conducted using R language software version 3.4.3 and Empower(R) software version 4.0. Statistical significance was defined as P values below 0.05 (two-sided). Baseline indicators were categorized based on the quartiles of TyG-BMI, and a comparison of the baseline characteristics was made for individuals in each group. Continuous variables were presented as median (interquartile range) or mean (SD: standard deviation), while categorical variables were described using percentages and frequencies. Differences in categorical variables between TyG-BMI groups were assessed using the χ2 test, and differences in continuous variables were examined using analysis of variance (ANOVA) and the Kruskal-Wallis H test.

Univariate and multivariate Cox regression analyses were employed to evaluate the relationship between TyG-BMI and the reversal of Pre-DM to normoglycemia. Four models were utilized: Model I (not adjusted for any covariates), Model II (adjusted for sex, age), Model III (adjusted for variables including AST, age, HDL-c, drinking status, DBP, family history of diabetes, ALT, SBP, Scr, LDL-c, sex, and smoking status). Significantly, the variable TC was omitted from the ultimate multivariate Cox proportional hazards regression equation as a result of collinearity with other variables (**Supplementary Table S1**). Additionally, the presence of patients diagnosed with DM at follow-up may hinder the observation of the reversal of Pre-DM to normoglycemia or potentially influence the probability of such an event occurring (29). Therefore, as described by Fine and Gray, competing risk multivariate Cox proportional hazards regression was used to validate the association between TyG-BMI and reversal of Pre-DM to normoglycemia (29, 30). This approach treated the progression of diabetes as a competing risk in relation to the occurrence of normoglycemic events.

In addition, the Cox proportional hazards regression model with cubic spline functions was employed to investigate the potential nonlinear relationship between TyG-BMI and the probability of returning to normoglycemia among participants with Pre-DM. In cases where nonlinearity was detected, inflection points were determined using a recursive algorithm. Subsequently, a two-part Cox proportional hazards regression model was constructed on each side of the identified inflection point. The best Model explaining the relationship between TyG-BMI and return to normoglycemia was ultimately screened by a log-likelihood ratio test.

Due to excessive missing information in variables such as smoking and drinking, there may be a bias. We used raw data without multiple imputations to explore the relationship between TyG-BMI and the probability of reverting from Pre-DM to normoglycemia to verify the reliability of the analysis obtained after multiple imputations. Besides, previous studies have indicated significant associations between glucose metabolism and smoking, family history of DM, and drinking (31–33). To validate the results, several sensitivity analyses were performed. Firstly, the analysis was performed on individuals who had never consumed alcohol (n=21,010). Additionally, individuals with a family history of DM were excluded from the sensitivity analysis (n=25,244). Furthermore, the relationship between TyG-BMI and return to normoglycemia from Pre-DM in never-smoking individuals was explored (n=15,858). Moreover, continuous covariates were integrated into the equation using generalized additive modeling (GAM). E-values were calculated to assess the likelihood of unmeasured confounding between TyG-BMI and regression from Pre-DM to normoglycemia (34).

Subgroup analyses of various subgroups (age, sex, SBP, HDL-c, LDL-c) were conducted using stratified Cox proportional hazards regression models. To accomplish this, continuous variables such as SBP and HDL-c were transformed into categorical variables based on clinical cutoff points (age: <30, 30- 40, 40 -50, 50- 60, 60 - 70, ≥70 years; HDL-c<1.0, HDL-c≥1.0 mmol/L; SBP: <140, ≥140 mmHg; LDL-c<2.5, LDL-c≥2.5 mmol/L) (35–37). Apart from the stratification factors, we made adjustments for age, smoking status, LDL-c, ALT, DBP, drinking status, SBP, sex, AST, HDL-c, BUN, and family history of diabetes. To assess the presence of interaction terms, likelihood ratio tests were employed in models with and without such terms.

## Results

### Characteristics of participants

**Figure 2:**
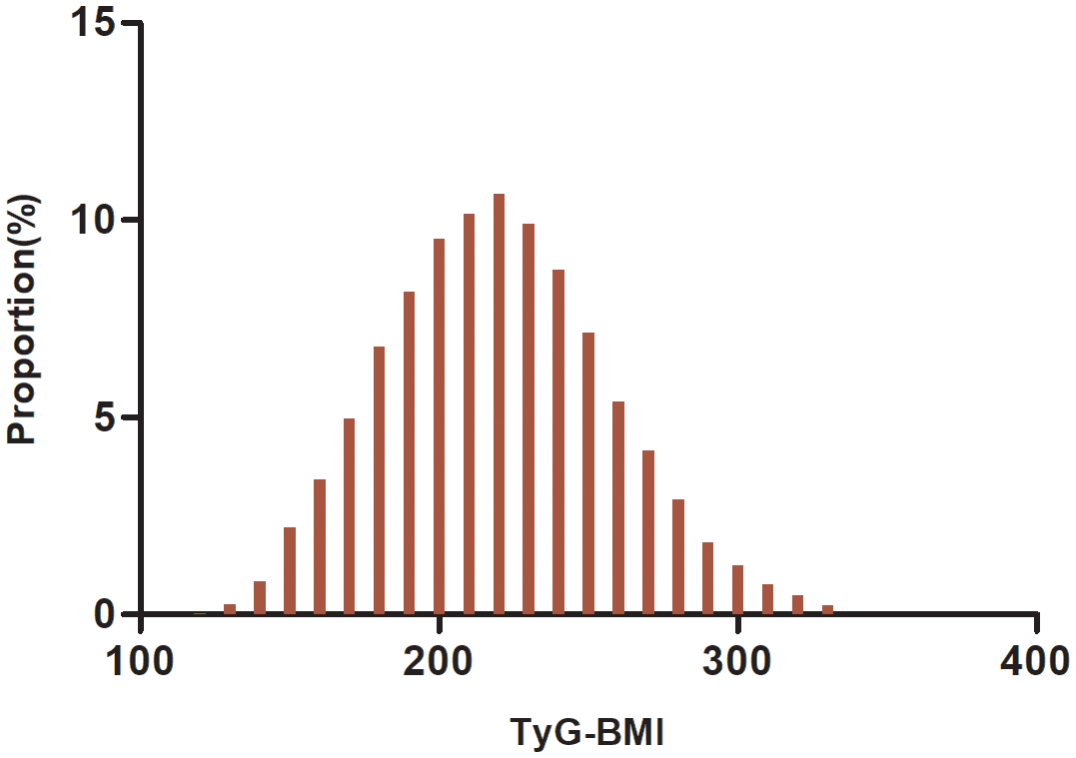
Distribution of TyG-BMI. It has a mean of 219.47 and a normal distribution with values between 116.94 and 334.08.

A total of 24,279 participants were included in the analysis, including 16,734 males and 8,545 females, whose mean age was 49.29 (13.82) years. The TyG-BMI was normally distributed from 116. 94 to 334.08, with a mean value of 219.47 (**Figure 2**). The anthropometric and biochemical characteristics of the patients, stratified according to TyG-BMI quartiles, are presented in **Table 1**. The results clearly demonstrate that various parameters such as LDL-c, age, height, DBP, weight, SBP, BMI, ALT, TyG, TC, AST, BUN, TG, TyG-BMI, and Scr exhibited a significant and gradual increase with higher TyG-BMI values. Conversely, HDL-c demonstrated an opposite trend. In addition, the proportions of males, drinkers, and smokers proportionally increased progressively with increasing TyG-BMI, while the proportion of females progressively decreased.

**Table 1:** The Baseline Characteristics of the Enrolled Participants.

TyG-BMI quartile	Q1 (<193.10)	Q2 (193.10-218.22)	Q3 (218.22-244.05)	Q4 ((≥244.04)	*P*-value
Participants(n)	6319	6320	6320	6320	
Age(years)	45.25 ± 14.30	50.29 ± 13.80	51.63 ± 13.34	50.00 ± 12.96	<0.001
Height(cm)	165.18 ± 8.29	166.32 ± 8.40	167.17 ± 8.25	168.06 ± 8.16	<0.001
Weight(kg)	57.30 ± 7.40	65.93 ± 7.50	71.97 ± 7.96	81.05 ± 9.93	<0.001
BMI (kg/m2)	20.94 ± 1.66	23.77 ± 1.28	25.69 ± 1.39	28.63 ± 2.21	<0.001
SBP (mmHg)	120.70 ± 16.57	126.40 ± 17.06	129.17 ± 17.32	132.40 ± 17.26	<0.001
DBP (mmHg)	73.91 ± 10.09	77.40 ± 10.60	79.76 ± 10.86	82.44 ± 11.17	<0.001
FPG (mmol/L)	5.86 ± 0.27	5.92 ± 0.30	5.97 ± 0.33	6.03 ± 0.34	<0.001
TC (mmol/L)	4.65 ± 0.88	4.93 ± 0.93	5.07 ± 0.92	5.25 ± 0.99	<0.001
TG (mmol/L)	0.84 (0.63-1.11)	1.24 (0.96-1.65)	1.67 (1.25-2.24)	2.33 (1.70-3.34)	<0.001
HDL-c(mmol/L)	1.44 ± 0.30	1.35 ± 0.30	1.29 ± 0.28	1.25 ± 0.29	<0.001
TyG	8.28 ± 0.43	8.69 ± 0.43	8.99 ± 0.46	9.38 ± 0.55	<0.001
LDL-c(mmol/L)	2.69 ± 0.67	2.90 ± 0.71	2.96 ± 0.71	3.00 ± 0.76	<0.001
AST(U/L)	22.80 ± 8.91	24.89 ± 11.14	26.99 ± 10.60	30.91 ± 14.79	<0.001
BUN (mmol/L)	4.81 ± 1.25	5.00 ± 1.23	5.07 ± 1.26	5.07 ± 1.25	<0.001
ALT(U/L)	15.50 (12.00-21.22)	20.00 (15.00-27.83)	24.60 (18.00-35.52)	32.00 (22.30-48.40)	<0.001
Scr (μmol/L)	68.27 ± 15.38	72.44 ± 15.50	74.43 ± 16.41	75.80 ± 15.66	<0.001
Sex					<0.001
Male	3092 (48.93%)	4066 (64.34%)	4587 (72.58%)	4989 (78.94%)	
Female	3227 (51.07%)	2254 (35.66%)	1733 (27.42%)	1331 (21.06%)	
Smoking status					<0.001
Current smoker	813 (12.87%)	1349 (21.34%)	1615 (25.55%)	2001 (31.66%)	
Ever smoker	198 (3.13%)	232 (3.67%)	291 (4.60%)	287 (4.54%)	
Never smoker	5308 (84.00%)	4739 (74.98%)	4414 (69.84%)	4032 (63.80%)	
Drinking status					<0.001
Current drinker	104 (1.65%)	212 (3.35%)	241 (3.81%)	381 (6.03%)	
Ever drinker	648 (10.25%)	934 (14.78%)	1075 (17.01%)	1240 (19.62%)	
Never drinker	5567 (88.10%)	5174 (81.87%)	5004 (79.18%)	4699 (74.35%)	
Family history of diabetes					0.220
No	6177 (97.75%)	6148 (97.28%)	6175 (97.71%)	6154 (97.37%)	
Yes	142 (2.25%)	172 (2.72%)	145 (2.29%)	166 (2.63%)	

Continuous variables were summarized as mean (SD) or medians (quartile interval); categorical variables were displayed as percentage (%).

BUN, blood urea nitrogen; LDL-c, low-density lipid cholesterol; TyG, the triglyceride-glucose index; FPG, fasting plasma glucose; SBP, systolic blood pressure; TG triglyceride BMI, body mass index; TC, total cholesterol, ALT, alanine aminotransferase; TyG-BMI, triglyceride glucose-body mass index; DBP, diastolic blood pressure; HDL-c, high-density lipoprotein cholesterol; AST aspartate aminotransferase; Scr, serum creatinine.

### Reversal rate from prediabetes to normoglycemia

**Figure 3:**
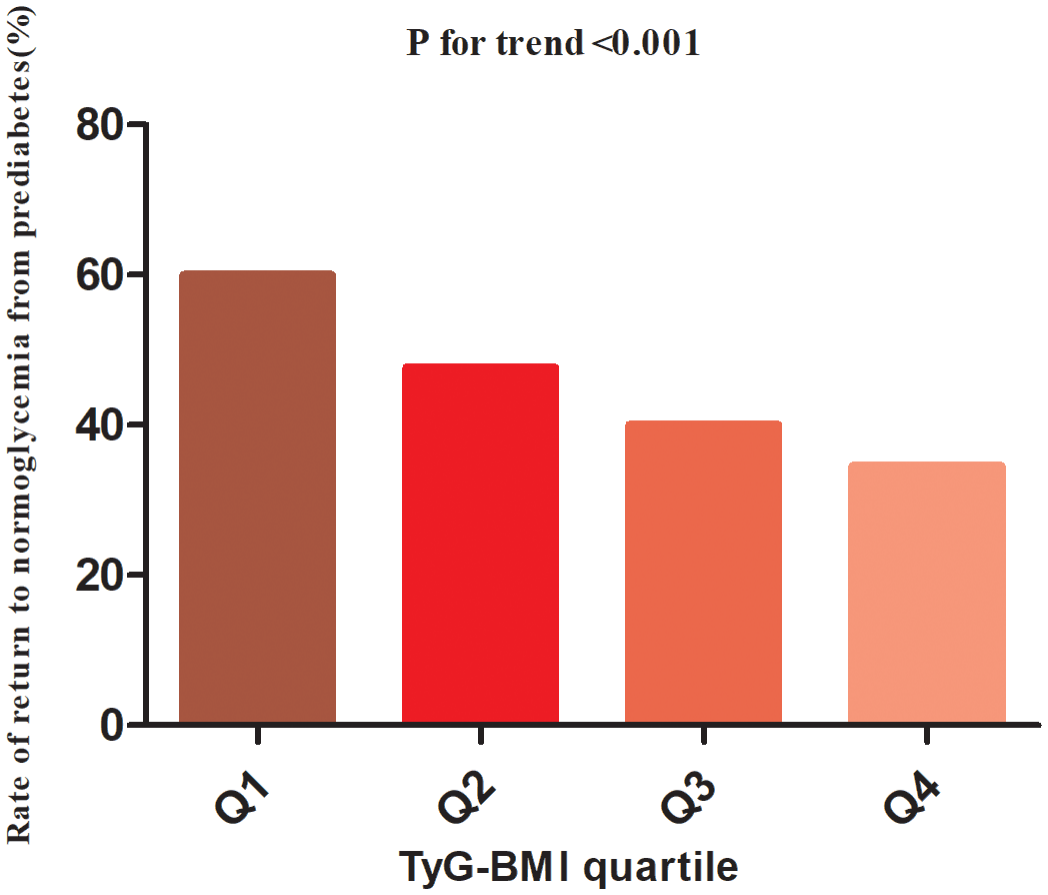
The rate of return to normoglycemia from prediabetes according to the quartiles of TyG-BMI. Participants with the highest TyG-BMI(Q4) had a lower regression rate to normoglycemia than those with the lowest TyG-BMI(Q1) (p<0.001 for trend).

During the minimum follow-up of 2 years, the maximum follow-up of 5 years, and the median follow-up time of 2.89 years, 11,511 patients with Pre-DM returned to normoglycemia. The overall rate of return to normoglycemia was 154.11/1000 person-years. Of these, the rates of return to normoglycemia in the TyG-BMI quartile group of participants were Q1: 206.01/1000 person-years, Q2: 162.12/1000 person-years, Q3: 134.52/1000 person-years and Q4: 115.81/1000 person-years, respectively. The overall reversal rate of prediabetes to normoglycemia was 45.54%. The reversal rates for each TyG-BMI quartile were Q1: 59.95%, Q2: 47.66%, Q3: 40.00%, Q4: 34.54% (**Figure 3**). The reversal rate was significantly lower in the higher participants with TyG-BMI than those with lower TyG-BMI (**Table 2** and **Figure 3**).

**Table 2:** Rate of return to normoglycemia from prediabetes (% or per 1000 person-years).

TyG-BMI	Participants (n)	Reversion events (n)	Reversal rate (95% CI) (%)	Per 1000 person-year
**Total**	25279	11511	45.54 (44.92-46.15)	154.11
**Q1(<193.08)**	6319	3788	59.95 (58.74-61.15)	206.01
**Q2 (193.08-218.21)**	6320	3012	47.66 (47.17-49.02)	162.12
**Q3 (218.21-244.04)**	6320	2528	40.00 (38.79-41.21)	134.52
**Q4 (≥244.04)**	6320	2183	34.54% (33.37-35.71)	115.81
***P* for trend**			<0.001	

TyG-BMI, triglyceride glucose-body mass index; CI, confidence interval.

### Univariate Cox proportional hazards regression was employed to analyze the relationship between the various influencing factors, including TyG-BMI, and the regression from Pre-DM to normoglycemia

The univariate analysis revealed significant negative associations between regression to normoglycemia and LDL-c, age, TG, AST, DBP, ALT, SBP, BUN, TC, BMI, and family history of diabetes, while positive associations were observed with HDL-c, never smoked, and never drinking alcohol (**Supplementary Table S2**).

**Figure 4:**
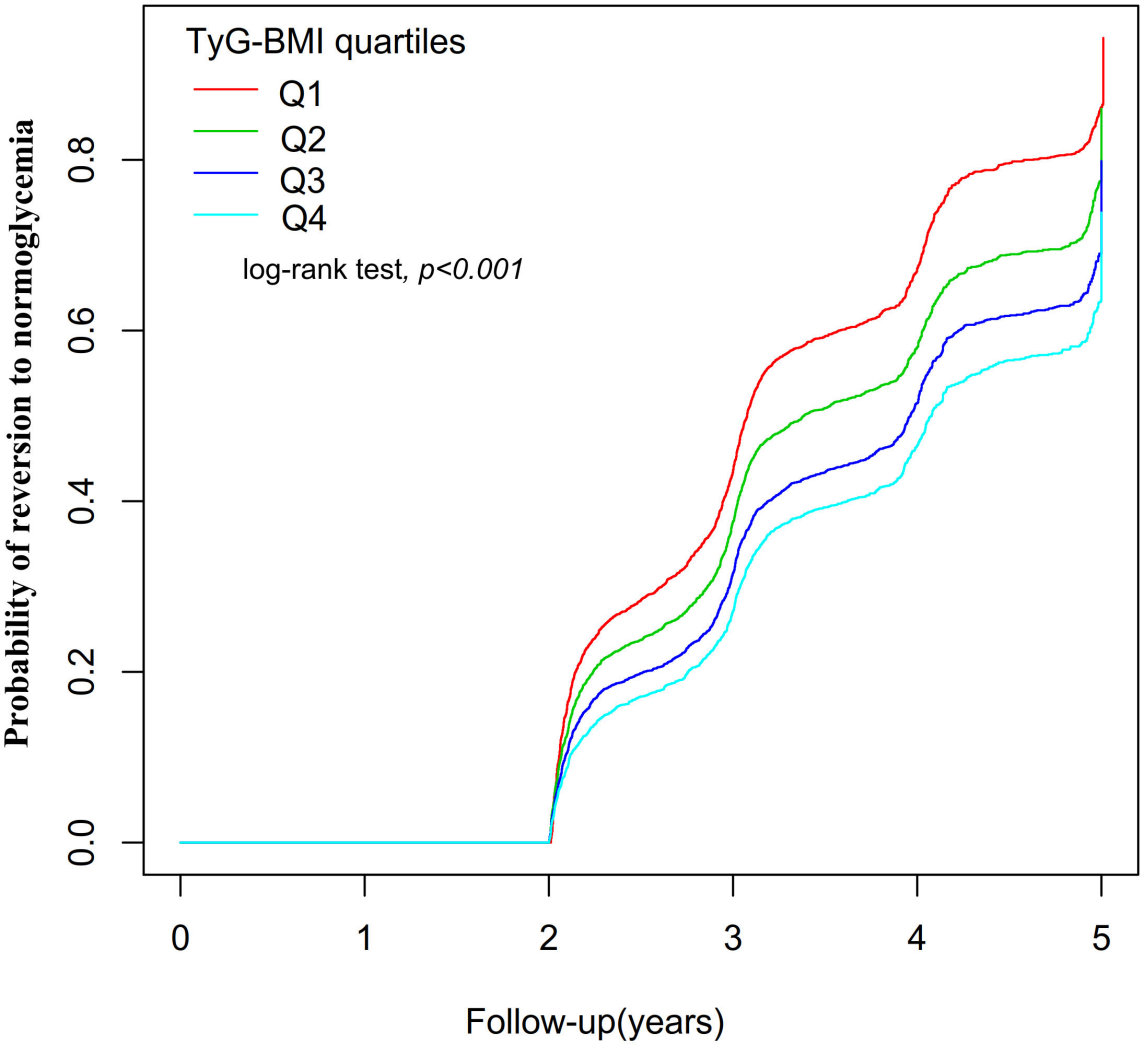
showed the Kaplan-Meier curves for the likelihood of transitioning from prediabetes to normoglycemia. Displayed the Kaplan-Meier curves illustrating the probability of regression from Pre-DM to normoglycemia based on TyG-BMI quartiles. There was a significant difference in the probability of returning to normoglycemia in the different TyG-BMI quartile groups (log-rank test, P<0.001). Compared with participants in the lowest TyG-BMI quartile group (Q1), participants in the higher TyG-BMI quartile groups (Q2 to Q4) had a lower probability of reversing to normoglycemia during the follow-up period.

**Figure 4** displayed the Kaplan-Meier curves illustrating the probability of regression from Pre-DM to normoglycemia based on TyG-BMI quartiles. There was a significant difference in the probability of returning to normoglycemia in the different TyG-BMI quartile groups (log-rank test, P<0.001). It can be seen that the cumulative reversal rate of the TyG-BMI quartile groups gradually increased with the duration of follow-up. In addition, compared with participants in the lowest TyG-BMI quartile group (Q1), participants in the higher TyG-BMI quartile groups (Q2 to Q4) had a lower probability of reversing to normoglycemia during the follow-up period. This indicated that individuals with the highest TyG-BMI had the lowest likelihood of reverting to normoglycemia from Pre-DM.

### Multivariate Cox proportional-hazards regression models to analyze the relationship between TyG-BMI and return to normoglycemia in patients with Pre-DM

Three Cox proportional-hazards regression models were constructed to explore the relationship between TyG-BMI and the probability of returning to normoglycemia from Pre-DM. In Model I, a 10-unit increase in TyG-BMI was related to a 6.7% reduction in the likelihood of normoglycemia (HR=0.933, 95% CI 0.928-0.938). After adjusting for demographic variables in Model II, each 10-unit increase in TyG-BMI was associated with a 4.8% decrease in the probability of regression to normoglycemia (HR=0.952, 95% CI 0.947-0.957). In Model III (fully adjusted Model), which accounted for various potential confounders, the HR for TyG-BMI (per 10 units) and the return to normoglycemia was 0.970 (95% CI: 0.965-0.976, p<0.001) (**Table 3**).

**Table 3:** Relationship between TyG-BMI and the probability of reversal to normoglycemia from prediabetes in different models.

Exposure	Model I (HR.,95%CI) *p*	Model II(HR,95%CI) *P*	Model III(HR,95%CI) *P*	Model IV(HR,95%CI) *P*
**TyG-BMI per 10 units**	0.933 (0.928, 0.938) <0.001	0.952 (0.947, 0.957) <0.001	0.970 (0.965, 0.976) <0.001	0.976 (0.970, 0.982) <0.001
TyG-BMI quartiles				
**Q1**	Ref	Ref	Ref	Ref
**Q2**	0.767 (0.731, 0.805) <0.001	0.882 (0.839, 0.926) <0.001	0.952 (0.905, 1.001) 0.056	0.981 (0.932, 1.033) 0.465
**Q3**	0.625 (0.594, 0.657) <0.001	0.753 (0.714, 0.793) <0.001	0.857 (0.811, 0.906) <0.001	0.899 (0.849, 0.953) <0.001
**Q4**	0.533 (0.506, 0.562) <0.001	0.629 (0.595, 0.664) <0.001	0.767 (0.720, 0.816) <0.001	0.805 (0.755, 0.858) <0.001
**P for trend**	<0.001	<0.001	<0.001	<0.001

Model I: we did not adjust other covariates.

Model II: we adjust sex age.

Model III: we adjust age, drinking status, sex, HDL-c, AST, DBP, Scr, ALT, family history of diabetes, LDL-c, SBP, and smoking status.

Model IV: we adjust age (smooth), drinking status, sex, DBP (smooth), ALT (smooth), HDL-c (smooth), AST (smooth), family history of diabetes, LDL-c (smooth), Scr (smooth), SBP (smooth), and smoking status.

HR, hazard ratio; Ref, reference; CI, confidence.

In addition, TyG-BMI was converted from a continuous variable to a categorical variable, and then the categorically converted TyG-BMI was reintroduced into the Cox proportional hazards regression model. The findings from the multivariable-adjusted Model showed that using the first quartile (Q1) of TyG-BM as a reference, the HRs of Q2, Q3, and Q4 in relation to the return to normoglycemia were 0.952 (0.905, 1.001), 0.857 (0.811, 0.906), and 0.767 (0.720, 0.816), respectively. In other words, the probability of returning to normoglycemia was 4.8% lower for participants in Q2, 14.3% lower for participants in Q3, and 23.3% lower for participants in Q4 compared to participants in Q1 (**Table 3**-Model III).

### Results of multivariate Cox proportional hazards regression for competing risks

Taking into account the development of prediabetes into diabetes as a competing event, the findings of the competing analyses are shown in **Table 4**. TyG-BMI and the likelihood of reverting to normoglycemia had a negative relationship in Model I (SHR=0.93, 95% CI: 0.93-0.94). SHR=0.95, 95% CI: 0.95-0.96 for Model II, which was sex- and age-adjusted, revealed consistent findings. The fully adjusted Model (Model III), which takes into consideration factors such as drinking status, AST, sex, DBP, HDL-c, ALT, age, BUN, family history of diabetes, Scr, TG, SBP, smoking status, and LDL-c, a negative connection between TyG-BMI and the return to normoglycemia was still seen (SHR=0.97, 95% CI: 0.96-0.98). When TyG-BMI was examined as a categorical variable, as well. The fully adjusted Model revealed that the likelihood of returning to normoglycemia decreased by 5%(SHR=0.95, 95% CI: 0.91-1.00) in the second quartile (Q2), by 14% in the third quartile(SHR=0.86, 95% CI: 0.81-0.91, and by 23% in the fourth quartile (SHR=0.77, 95% CI: 0.72, 0.82) when compared to participants in the first quartile (Q1).

**Table 4:** Relationship between TyG-BMI and reversion to normoglycemia in patients with prediabetes in different models with competing risk of progression to diabetes.

Exposure	Model I(SHR,95%CI, P)	Model II(SHR,95%CI, P)	Model III (SHR,95%CI, P)
**TyG-BMI per 10 units**	0.93 (0.93, 0.94) <0.001	0.95 (0.95, 0.96) <0.001	0.97 (0.96, 0.98) <0.001
TyG-BMI quartile			
**Q1**	Ref.	Ref.	Ref.
**Q2**	0.77 (0.73, 0.80) <0.001	0.88 (0.84, 0.93) <0.001	0.95 (0.91, 1.00) 0.056
**Q3**	0.62 (0.59, 0.66) <0.001	0.75 (0.71, 0.79) <0.001	0.86 (0.81, 0.91) <0.001
**Q4**	0.53 (0.51, 0.56) <0.001	0.63 (0.59, 0.66) <0.001	0.77 (0.72, 0.82) <0.001
**P for trend**	<0.001	<0.001	<0.001

Model I: we did not adjust other covariates.

Model II: we adjust age, sex.

Model III: we adjust age, drinking status, sex, HDL-c, AST, DBP, Scr, ALT, family history of diabetes, LDL-c, SBP, and smoking status.

SHR, subdistribution hazard ratios; Ref, reference; CI, confidence.

### Sensitivity analysis

A series of sensitivity analyses were conducted to ensure the robustness of our findings. The GAM was first used to introduce continuous covariates as curves into the equation. As depicted in **Table 3** (Model IV), the result was generally consistent with the fully adjusted Model. The HR between TyG-BMI (per 10 units) and the probability of returning to normoglycemia was found to be 0.976 (95% CI: 0.970, 0.982). In addition, we used raw data without multiple imputations to investigate the relationship between TyG-BMI and the probability of reverting from Pre-DM to normoglycemia. The results of the multivariate regression analysis showed that the HR (95% CI) for the association between TyG-BMI (per 10 units) and the probability of reverting to normoglycemia was 0.973 (0.952, 0.994), which is consistent with the results from the data analysis after multiple imputation (**Supplementary Table S3**).

A sensitivity analysis was performed on participants (n = 18,493) who had never smoked. After adjusting for confounding variables, the findings revealed that TyG-BMI was also found to be negatively associated with the probability of regression to normoglycemia from Pre-DM (HR=0.970, 95% CI: 0.963-0.977). Similarly, when patients with a family history of DM were excluded from the analysis (n=24,654), and after adjusting for confounding variables, the results showed that TyG-BMI remained negatively associated with return to normoglycemia in patients with Pre-DM (HR=0.971, 95% CI. 0.965-0.978). Furthermore, by restricting the analysis to patients who had never consumed alcohol, the Cox proportional hazards regression results showed the HR between TyG-BMI and the probability of returning to normoglycemia was 0.970 (95% CI: 0.964, 0.976) (**Table 5**).

**Table 5:** Association between TyG-BMI and reversion to normoglycemia in different sensitivity analyses.

Exposure	Model I (HR.,95%CI) *p*	Model I I(HR,95%CI) *p*	Model III(HR,95%CI) *p*
**TyG-BMI (per 10 units)**	0.970 (0.963, 0.977) <0.001	0.971 (0.965, 0.978) <0.001	0.970 (0.964, 0.976) <0.001
TyG-BMI quartiles			
**Q1**	Ref	Ref	Ref
**Q2**	0.964 (0.911, 1.019) 0.195	0.952 (0.901, 1.005) 0.076	0.956 (0.908, 1.006) 0.086
**Q3**	0.860 (0.806, 0.917) <0.001	0.837 (0.787, 0.891) <0.001	0.858 (0.810, 0.907) <0.001
**Q4**	0.764 (0.710, 0.822) <0.001	0.789 (0.736, 0.846) <0.001	0.765 (0.718, 0.815) <0.001
**P for ternd**	<0.001	<0.001	<0.001

Model I involved a sensitivity analysis with participants (N=18,493) who had never smoked. Age, drinking status, HDL-c, AST, sex, DBP, Scr, ALT, family history of diabetes, LDL-c, SBP, and smoking status were all adjusted.

Model II was a sensitivity analysis conducted on participants (N=20,444) who had not consumed alcohol. Age, HDL-c, sex, AST, DBP, Scr, ALT, family history of diabetes, LDL-c, and SBP were all adjusted.

Model III involved a sensitivity analysis after excluding participants with a family history of diabetes (N=24,654). Age, drinking status, sex, HDL-c, AST, DBP, Scr, ALT, LDL-c, SBP, and smoking status were all adjusted.

CI stands for “confidence interval,” and “Ref” stands for “reference”.

Additionally, when TyG-BMI was used as a categorical variable, sensitivity analyses of the multivariate-adjusted Model demonstrated that the probability of returning to normoglycemia from Pre-DM was significantly lower in patients in the third and fourth quartiles compared with participants in the first quartile of TyG-BMI (**Table 5**). Furthermore, the E-value (1.28) was found to be greater than the relative risk of TyG-BMI and unmeasured confounders (1.14), suggesting that unknown or unmeasured variables may have little effect on the relationship between TyG-BMI and return to normoglycemia from Pre-DM. Based on all sensitivity analyses, our findings were robust.

### Cox proportional hazards regression modeling using cubic spline functions to address nonlinearity

**Figure 5:**
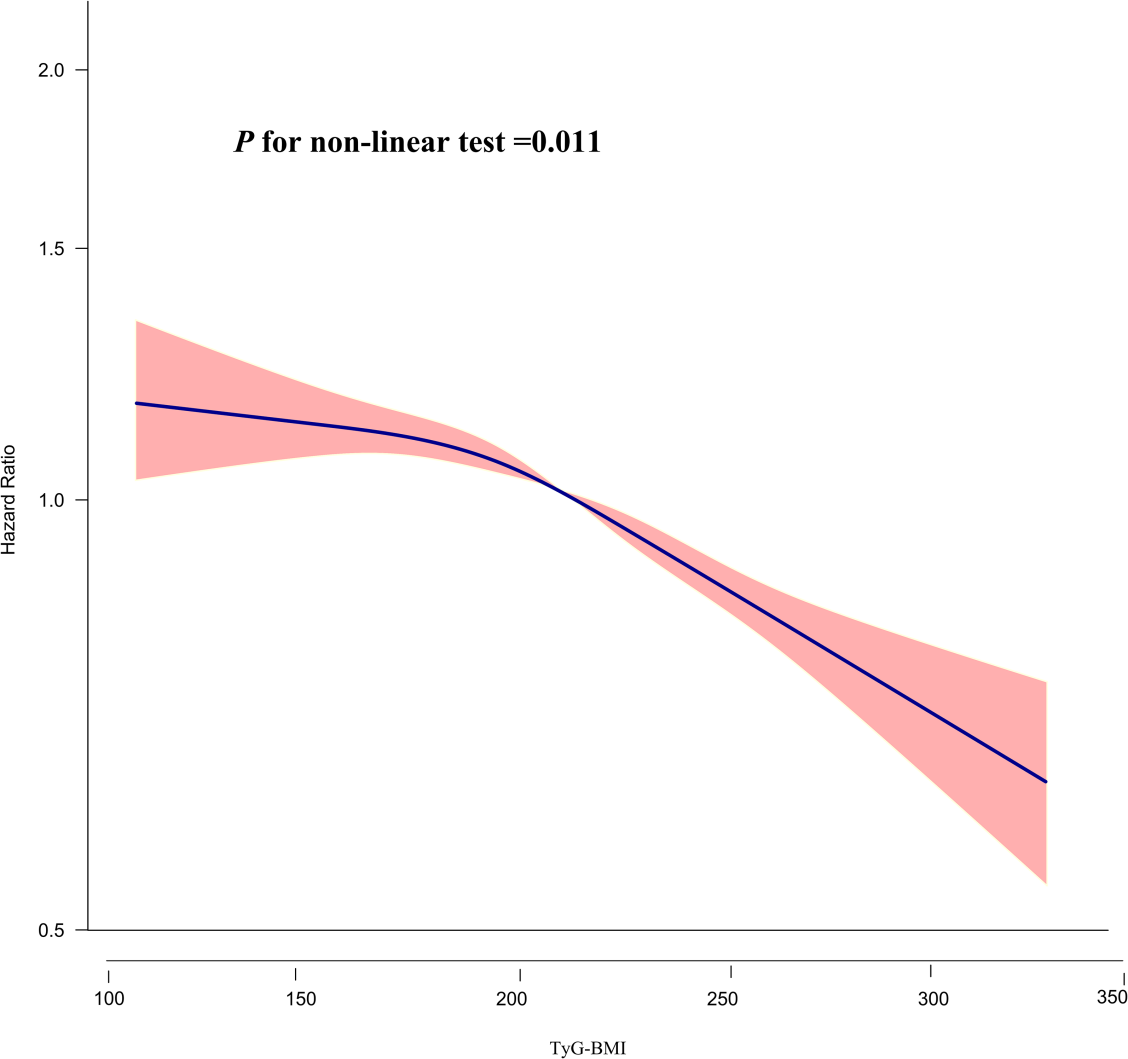
The nonlinear relationship between TyG-BMI and the probability of reversion to normoglycemia from Pre-DM. The result showed that the relationship between TyG-BMI and regression to normoglycemia from Pre-DM was nonlinear, with the inflection point of TyG-BMI being 196.46.

The relationship between TyG-BMI and the probability of returning to normoglycemia from Pre-DM was found to be nonlinear (P for nonlinear test=0.011) as determined by the Cox proportional hazards regression model with a cubic spline function (**Table 6**; **Figure 5**). Using the recursive technique, the TyG-BMI inflection point was identified at 196.46. Subsequently, HR and CI were obtained on either side of the inflection point using a two-piecewise Cox proportional hazards regression model. Before the inflection point, the HR was 0.993 (95% CI: 0.978, 1.008, P=0.372, per 10 units), which showed no statistically significant difference. However, after the inflection point, the HR was 0.962 (95% CI: 0.954, 0.970; per 10 units), indicating a significant negative association.

**Table 6:** The result of the two-piecewise linear regression model.

Outcome:	HR,95%CI *p*
**Standard Cox regression**	0.970 (0.965, 0.976) <0.0001
**Two-piecewise Cox regression**	196.46
**TyG-BMI<196.46 (per 10 units)**	0.993 (0.978, 1.008) 0.372
**TyG-BMI≥196.46 (per 10 units)**	0.962 (0.954, 0.970) <0.001
**P for log-likelihood ratio test**	0.001

Adjusted covariates included age, drinking status, sex, HDL-c, DBP, AST, Scr, ALT, LDL-c, family history of diabetes, SBP, and smoking status.

### Results of subgroup analysis

The link between TyG-BMI and reversion to normoglycemia from Pre-DM was not affected by sex, age, DBP, SBP, HDL-C, or LDL-C in any of the prespecified or exploratory subgroups examined (**Supplementary Table S4**). That is to say, the interaction between these variables and TyG-BMI was not statistically significant (P > 0.05 for interaction).

## Discussion

This large retrospective cohort study demonstrated that the probability of reversion to normoglycemia in patients with Pre-DM was negatively associated with TyG-BMI levels. Additionally, a threshold effect curve was identified, with an inflection point of 196 for TyG-BMI; on either side of the inflection point, different associations between TyG-BMI and regression to normoglycemia were observed.

In one study, it was found that within one year of follow-up, 54% of people with Pre-DM had regression to normoglycemia, and 6% progressed to DM (38). Another prospective cohort study, which included 491 participants, showed that during a median follow-up of 2.5 years, the rate of return to normoglycemia from Pre-DM was 22.6% (39). Additionally, in another cohort study from China that included 14,231 adults with Pre-DM, 44.9% of them had regression to normoglycemia within 2 years (12). In our study, at a median follow-up time of 2.95 years, the rate of reversal of Pre-DM to normoglycemia was 45.54%. The rate of return to normoglycemia in patients with Pre-DM varied across studies, which may be attributed to differences in participant age, ethnicity, and duration of follow-up. It is important to emphasize that a significant proportion of patients with Pre-DM can return to normoglycemia, as confirmed by all studies. Therefore, identifying the triggers for the regression to normoglycemia in patients with Pre-DM is particularly important for the prevention of diabetes and its complications.

The triglyceride-glucose (TyG) index was first reported in 2008 and was considered to be a reliable, inexpensive, and simple alternative to insulin resistance (40–42). More recently, TyG-BMI (an index that combines BMI and TyG) has been recognized as a more reliable identifier of IR than TyG (16). In addition, several recent studies have demonstrated a strong association between TyG-BMI and stroke, NAFLD, hypertensive disease, and DM (43–45). A cohort study conducted in a Chinese general population revealed that TyG-BMI was independently associated with the risk of new-onset diabetes after excluding other confounders (HR 1.50 per SD increase, 95% CI: 1.40-1.60) (21). This conclusion was reinforced by a cross-sectional study carried out in Spain, where participants in the fourth quartile of TyG-BMI had a 3.63-fold higher chance of getting diabetes than those in the first quartile (OR=4.63, 95%CI:3.12-6.89) (46). Additional studies have demonstrated that TyG-BMI is also independently and positively associated with the risk of Pre-DM (20, 47). Hence, we postulated that TyG-BMI may exhibit a negative association with the likelihood of reversion to normoglycemia from Pre-DM. However, the majority of current studies have focused on TyG-BMI and disease progression, neglecting to explore the link between TyG-BMI and the regression of Pre-DM to normoglycemia. Our study supports the hypothesis that elevated TyG-BMI is related to a decreased probability of return to normoglycemia in patients with Pre-DM. Identifying TyG-BMI as a risk factor for return to normoglycemia and elucidating the association between them will provide valuable insights for achieving normoglycemia in patients with Pre-DM and for promoting the prevention of DM and its complications. Additionally, this study used TyG-BMI as both a categorical and continuous variable to explore its relationship with regression to normoglycemia in patients with Pre-DM, thereby minimizing information loss and quantifying the relationship. Moreover, sensitivity analyses specifically focused on participants who reported never smoking, having no family history of diabetes, and abstaining from alcohol consumption. The results of sensitivity analyses further support the existence of the aforementioned relationship in this subgroup of participants. Besides, competing risks multivariate Cox regression analysis yielded consistent results with the multivariate Cox proportional hazards regression model regarding the relationship between TyG-BMI and return to normoglycemia from Pre-DM. These results validate the stability of our findings.

In clinical practice, this study emphasizes the potential utility of TyG-BMI as a simple, low-cost stratification tool to identify high-risk individuals among Chinese adults with Pre-DM. Individuals with a higher TyG-BMI have an increased risk of either remaining prediabetic or developing DM, with a smaller likelihood of reverting to normoglycemia. This can assist clinicians in prioritizing interventions for high-risk groups. Specifically, this includes:(1)Targeted Interventions: For patients with elevated TyG-BMI, clinicians might consider adopting more aggressive lifestyle intervention measures, such as dietary modifications, increased physical exercise, and a management plan targeting triglycerides, glucose metabolism, and weight. (2) Monitoring and Follow-up: Patients with a higher TyG-BMI may require more frequent monitoring to prevent the onset of DM. Regular follow-ups can help control and potentially reverse their prediabetic state. (3) Patient Education: Educating patients about the importance of TyG-BMI as an indicator of their health status can motivate them to adhere to the prescribed lifestyle adjustments and treatment methods. Understanding the relationship between TG, blood glucose, and weight and their health outcomes can encourage patients to participate in their care. (4) Clinical Guidelines: The defined relationship can be incorporated into clinical guidelines for the management of Pre-DM. (5) Pharmacological Interventions: In certain situations, particularly when lifestyle changes fail to yield significant results or when TyG-BMI is particularly high, clinicians might consider pharmacological interventions to control triglyceride levels and glucose metabolism. Overall, the study’s findings offer valuable insights into potential interventions for prediabetic patients to promote their return to normoglycemia and reduce the risk of developing diabetes, ultimately contributing to improved public health and better management of Pre-DM.

In addition, a nonlinear relationship between TyG-BMI and reversion to normoglycemia in adults with Pre-DM was observed for the first time in our study. The inflection point of TyG-BMI was determined to be 196.46. When TyG-BMI was greater than 196.46, the probability of reversion to normoglycemia increased by 3.8% for each 10-unit decrease in TyG-BMI. On the other hand, when TyG-BMI was less than 196.46, there was no significant link between them. In other words, as the TyG-BMI of patients decreased, the probability of reversion to normoglycemia gradually increased. However, when the TyG-BMI decreased to about 196.46, the probability did not continue to increase but remained stable. The finding of a curvilinear relationship between TyG-BMI and reversion to normoglycemia is of great clinical value. It facilitates clinical consultation and informs decision-making to optimize DM prevention. People with Pre-DM are not only at higher risk for DM but also for cardiovascular disease and all-cause mortality. Previous studies have shown that even a brief return to normoglycemia significantly reduces the risk of DM in patients with Pre-DM. Therefore, the goal of treatment for Pre-DM should be to restore normoglycemia rather than merely prevent Pre-DM from developing into DM. Lifestyle interventions, encompassing dietary modifications and physical activity, have been demonstrated to be useful in preventing and managing pre-DM and DM (4, 48, 49). Our study determined the TyG-BMI threshold for restoring normoglycemia in Chinese patients with Pre-DM. In other words, the probability of return to normoglycemia can be significantly increased by dietary interventions and lifestyle changes, combined reduction of BMI and TG, and keeping TyG-BMI as close as possible to about 196.46.

This study possesses several noteworthy strengths: (i) The relationship between TyG-BMI and the reversal of Pre-DM to normoglycemia was explored for the first time using a study population of Chinese prediabetic patients. (ii) The sample size of this study exceeded 20,000, encompassing multiple regions in China, rendering the findings relatively objective and applicable to the Chinese population. (iii) The study revealed the nonlinear link between TyG-BMI and the reversal of Pre-DM to normoglycemia and was successful in pinpointing the inflection point. It’s wonderful progress. (iv) Missing data were accounted for using multiple imputations, maximizing statistical power while minimizing bias arising from missing data on covariates. (v) To ensure the robustness of the findings, a series of sensitivity analyses were performed, including converting TyG-BMI to a categorical variable, employing generalized additive modeling to insert continuous covariates as curves into the equation, utilizing competing risk models, and reanalyzing the association between TyG-BMI and regression to normoglycemia after excluding drinkers, smokers, and participants with a family history of DM.

However, it is important to acknowledge the following limitations: (i) The study population consisted solely of Chinese participants, warranting the need for further investigations to assess the association between the new risk marker, TyG-BMI, and the probability of reversing prediabetes to normoglycemia in diverse populations with different genetic backgrounds. In the future, collaborations with researchers outside of China are planned to validate the association between them in populations with diverse genetic backgrounds. (ii) Pre-DM was defined based on FPG levels of 5.6-6.9 mmol/l, without considering glycated hemoglobin measurements (HbA1C) or a 2-hour oral glucose tolerance test (2h-OGTT). Consequently, the screening of the population with Pre-DM may have been underestimated. However, collecting HbA1c measurements or conducting 2h-OGTT for such a large cohort is challenging. In addition, we used the FPG at the last follow-up as the criterion for reversal to normoglycemia, which may underestimate the rate of reversal to normoglycemia in patients with Pre-DM and lead to biased results. However, exploring the relationship between them in the context of underestimating the rate of reversion to normoglycemia from Pre-DM is more inclined to yield negative results. Nonetheless, we still uncovered an independent relationship between TyG-BMI and reversion to normoglycemia, further confirming the actual existence of an association between them. In the future, we will try to increase the number of follow-up visits and collect more information about 2-hour plasma glucose and HBA1C to make the results more reliable. (iii) Adjustments for factors such as waist circumference, waist-to-hip ratio, and insulin concentration were limited because the study was a secondary analysis of published data. However, E-values were computed to evaluate the possible impact of unmeasured confounders, and it was concluded that these confounders were unlikely to account for the study results. (iv) The investigation only assessed TyG-BMI and other parameters at baseline and did not include an analysis of changes in TyG-BMI over time. Future efforts will involve constructing new studies or collaborating with other researchers to collect information on various factors, including changes in TyG-BMI over time. Lastly, it is essential to highlight that this retrospective observational study can only suggest an independent link between TyG-BMI and the reversal to normoglycemia in patients with Pre-DM and does not confirm a causal association.

## Conclusion

This study demonstrated a negative and nonlinear relationship between TyG-BMI and reversal to normoglycemia in Chinese adults with prediabetes. When TyG-BMI was ≥196.46, it was significantly and negatively associated with the probability of reversal to normoglycemia in prediabetes. Clinicians and patients can work together, reducing TyG-BM to 196.46 in patients with Pre-DM by combining dietary interventions and lifestyle changes to reduce BMI and TG can significantly increase the probability of successfully reverting to normoglycemia.

## Data availability statement

The original contributions presented in the study are included in the article/**Supplementary Material**, further inquiries can be directed to the corresponding author/s.

## Ethics statement

The studies involving humans were approved by The Rich Healthcare Group Review Board. The studies were conducted in accordance with the local legislation and institutional requirements. The ethics committee/institutional review board waived the requirement of written informed consent for participation from the participants or the participants’ legal guardians/next of kin because Due to the study’s retrospective nature, the institutional ethics committee (The Rich Healthcare Group Review Board) waived the requirement for informed consent and approval.

## Author contributions

YS: Data curation, Formal analysis, Writing – original draft. HH: Methodology, Software, Writing – original draft. CC: Data curation, Methodology, Software, Writing – original draft. YH: Methodology, Software, Supervision, Validation, Writing – review & editing. CW: Software, Validation, Writing – review & editing.
