# Supplementary material for: Elevated triglyceride-glucose-body mass index associated with lower probability of future regression to normoglycemia in Chinese adults with prediabetes: a 5-year cohort study
Source: Front Endocrinol (Lausanne). 2024 Feb 13;15:1278239. doi: 10.3389/fendo.2024.1278239 (PMC10898590; doi:10.3389/fendo.2024.1278239)
Supplement: Supplementary file 1 [file Table_1.docx]

**Elevated Triglyceride-Glucose-Body Mass Index Associated with Lower Probability of Future Regression to Normoglycemia in Chinese Adults with Prediabetes: A 5-Year Cohort Study**

**Running title: TyG-BMI** and **normoglycemia**

Yang Shao ^1,2#^, Haofei Hu ^3#^, Changchun Cao^4#^ , Yong Han^5*^, Cen Wu ^6*^.

^1^ Department of Laboratory Medicine, Shengjing Hospital of China Medical University, Shenyang 110004, Liaoning Province,China.

^2^ Liaoning Clinical Research Center for Laboratory Medicine, Shenyang 110004, Liaoning Province,China.

^3^ Department of Nephrology, Shenzhen Second People's Hospital, Shenzhen 518000, Guangdong Province, China.

^4^ Department of Rehabilitation, Shenzhen Dapeng New District Nan'ao People's Hospital, Shenzhen 518000, Guangdong Province, China

^5^ Department of Emergency, Shenzhen Second People's Hospital, Shenzhen 518000, Guangdong Province, China.

^6^ Department of Pulmonary and Critical Care Medicine, Shengjing Hospital of China Medical University, Shenyang 110004, Liaoning Province,China.

^#^ Shao Yang, Haofei Hu and Changchun Cao have contributed equally to this work.

***Corresponding author**

Yong Han

Department of Emergency,

Shenzhen Second People’s Hospital,

No.3002 Sungang Road, Futian District,

Shenzhen 518000,

Guangdong Province,

China

Hanyong511023@163.com

***Corresponding author**

Cen Wu

Department of Pulmonary and Critical Care Medicine,

Shengjing Hospital of China Medical University,

NO.36, Sanhao Street, Heping District,

Shenyang 110004,

Liaoning Province,

China.

wuc@sj-hospital.org

Table S1 Collinearity screening

|  | Step 1 | Step 2 |
| --- | --- | --- |
| BMI (kg/m^2^) | 1.3 | 1.3 |
| Age(years) | 1.4 | 1.4 |
| Sex | 1.9 | 1.9 |
| SBP (mmHg) | 1.9 | 1.9 |
| DBP (mmHg) | 1.8 | 1.8 |
| TC (mmol/L) | 6.4 | NA |
| TG (mmol/L) | 1.9 | 1.2 |
| HDL-c(mmol/L) | 1.4 | 1.2 |
| LDL-c(mmol/L) | 5.3 | 1.1 |
| ALT(U/L) | 3.5 | 3.5 |
| ALT(U/L) | 3.2 | 3.2 |
| BUN (mmol/L) | 1.2 | 1.2 |
| Scr (μmol/L) | 1.8 | 1.8 |
| Drinking status | 1.1 | 1.1 |
| Family history of diabetes | 1 | 1 |
| Smoking status | 1.2 | 1.2 |

Variables excluded from collinearity screening: TC

Abbreviations: DBP, diastolic blood pressure; BMI, body mass index; TC, total cholesterol, SBP, systolic blood pressure; TG triglyceride, BMI, body mass index; AST aspartate aminotransferase; LDL-c, low-density lipid cholesterol; ALT, alanine aminotransferase; BUN, blood urea nitrogen; HDL-c, high-density lipoprotein cholesterol; Scr, serum creatinine.

Table S2. Factors influencing the probability of regression to **normoglycemia** from prediabetes analyzed by univariate Cox proportional hazards regression.

|  | Statistics | HR (95%CI) P value |
| --- | --- | --- |
| Age(years) | 49.292 ± 13.819 | 0.977 (0.975, 0.978) <0.001 |
| SEX |  |  |
| Male | 16734 (66.197%) | Ref |
| Female | 8545 (33.803%) | 1.259 (1.213, 1.308) <0.001 |
| Height(cm) | 166.684 ± 8.343 | 0.998 (0.996, 1.000) 0.115 |
| Weight(kg) | 69.063 ± 11.972 | 0.986 (0.984, 0.987) <0.001 |
| SBP (mmHg) | 127.168 ± 17.586 | 0.990 (0.988, 0.991) <0.001 |
| DBP (mmHg) | 78.377 ± 11.135 | 0.985 (0.983, 0.987) <0.001 |
| TC (mmol/L) | 4.975 ± 0.957 | 0.880 (0.863, 0.897) <0.001 |
| BMI (kg/m2) | 24.758 ± 3.265 | 0.935 (0.930, 0.941) <0.001 |
| FPG (mmol/L) | 5.945 ± 0.317 | 0.223 (0.207, 0.240) <0.001 |
| TG (mmol/L) | 1.778 ± 1.432 | 0.890 (0.876, 0.904) <0.001 |
| HDL-c(mmol/L) | 1.333 ± 0.303 | 1.615 (1.524, 1.711) <0.001 |
| TyG | 8.836 ± 0.619 | 0.705 (0.684, 0.727) <0.001 |
| TyG-BMI | 219.480 ± 37.053 | 0.993 (0.993, 0.994) <0.001 |
| LDL-c(mmol/L) | 2.885 ± 0.723 | 0.907 (0.884, 0.931) <0.001 |
| ALT(U/L) | 28.288 ± 23.216 | 0.993 (0.992, 0.994) <0.001 |
| AST(U/L) | 26.400 ± 11.939 | 0.988 (0.986, 0.989) <0.001 |
| BUN (mmol/L) | 4.990 ± 1.251 | 0.953 (0.939, 0.968) <0.001 |
| Scr (μmol/L) | 72.735 ± 15.996 | 0.997 (0.996, 0.998) <0.001 |
| Smoking status |  |  |
| Current smoker | 5778 (22.857%) | Ref |
| Ever smoker | 1008 (3.987%) | 1.192 (1.078, 1.318) <0.001 |
| Never smoker | 18493 (73.156%) | 1.290 (1.232, 1.350) <0.001 |
| Drinking status |  |  |
| Current drinker | 938 (3.711%) | Ref |
| Ever drinker | 3897 (15.416%) | 1.195 (1.065, 1.340) <0.001 |
| Never drinker | 20444 (80.873%) | 1.315 (1.182, 1.463) <0.001 |
| Family history of diabetes |  |  |
| No | 24654 (97.528%) | Ref |
| Yes | 625 (2.472%) | 0.761 (0.672, 0.861) <0.001 |

Continuous variables were summarized as mean (SD) or medians (quartile interval); categorical variables were displayed as percentage (%)

Abbreviations: FPG, fasting plasma glucose; BUN, blood urea nitrogen; BMI, body mass index; SBP, systolic blood pressure; TyG, the triglyceride-glucose index; TG triglyceride, DBP, diastolic blood pressure; TC, total cholesterol, ALT, alanine aminotransferase; TyG-BMI, triglyceride glucose-body mass index; LDL-c,low-density lipid cholesterol; AST aspartate aminotransferase; HDL-c, high-density lipoprotein cholesterol; Scr, serum creatinine.

Table S3. Relationship between TyG-BMI and the probability of reversal to normoglycemia from prediabetes in different models(Raw data without multiple imputation)

| Exposure | Model I (HR.,95%CI) *p* | Model II (HR.,95%CI) *p* | Model III (HR.,95%CI) *p* |
| --- | --- | --- | --- |
| TyG-BMI per 10 units | 0.933 (0.929, 0.938) <0.00001 | 0.952 (0.947, 0.957) <0.00001 | 0.973 (0.952, 0.994) 0.01385 |
| TyG-BMI quartiles |  |  |  |
| Q1 | Ref | Ref | Ref |
| Q2 | 0.772 (0.736, 0.810) <0.00001 | 0.887 (0.844, 0.931) <0.00001 | 0.750 (0.627, 0.897) 0.00166 |
| Q3 | 0.629 (0.598, 0.661) <0.00001 | 0.756 (0.717, 0.797) <0.00001 | 0.787 (0.646, 0.958) 0.01671 |
| Q4 | 0.535 (0.507, 0.564) <0.00001 | 0.630 (0.596, 0.666) <0.00001 | 0.696 (0.560, 0.865) 0.00111 |

Model I: we did not adjust other covariates.

Model II: we adjust sex age.

Model III: we adjust age, drinking status, sex, HDL-c, AST, DBP, Scr, ALT, family history of diabetes, LDL-c, SBP, and smoking status.

**Table S4. Stratified associations between TyG-BMI and reversion to normoglycemia in patients with prediabetes by age, sex, SBP, DBP, LDL-c, and HDL-c.**

| Characteristic | No of participants | HR (95%CI) P value P for interaction |
| --- | --- | --- |
| Age(years)  <30  30 to <40  40 to <50  50 to <60  60 to <70 | 1464  6055  5514  5907  4278 | 0.1394  0.982 (0.963, 1.000) 0.0519  0.981 (0.971, 0.992) 0.0005  0.969 (0.957, 0.982) <0.0001  0.964 (0.951, 0.977) <0.0001  0.951 (0.936, 0.966) <0.0001 |
| ≥70 | 2061 | 0.958 (0.936, 0.980) 0.0003 |
| Sex |  | 0.2952 |
| Male | 16734 | 0.967 (0.960, 0.974) <0.0001 |
| Female | 8545 | 0.977 (0.968, 0.986) <0.0001 |
| SBP (mmHg) |  | 0.3673 |
| <140 | 19904 | 0.970 (0.963, 0.976) <0.0001 |
| ≥140 | 5375 | 0.973 (0.960, 0.986) <0.0001 |
| DBP (mmHg) |  | 0.2505 |
| <90 | 965 | 0.934 (0.928, 0.939) <0.0001 |
| ≥90 | 3899 | 0.962 (0.947, 0.977) <0.0001 |
| HDL-c |  | 0.1824 |
| <1.0mmol/l | 3169 | 0.932 (0.918, 0.947) <0.0001 |
| ≥1.0 mmol/l | 22110 | 0.937 (0.932, 0.942) <0.0001 |
| LDL-c |  | 0.4775 |
| <2.5 mmol/l | 7715 | 0.923 (0.915, 0.931) <0.0001 |
| ≥2.5 mmol/l | 17564 | 0.940 (0.934, 0.946) <0.0001 |

Note 1: Above model adjusted for age, drinking status, sex, HDL-c, DBP AST, Scr, ALT, LDL-c, family history of diabetes SBP, and smoking status.

Note 2: In each case, the model is not adjusted for the stratification variable.

HR, Hazard ratios; CI: confidence, Ref: reference.
